# Smart cities and innovative urban management: Perspectives of integrated technological solutions in urban environments

**DOI:** 10.1016/j.heliyon.2024.e27850

**Published:** 2024-03-15

**Authors:** Elizeu Jacques, Alvaro Neuenfeldt Júnior, Sabine De Paris, Matheus Francescatto, Julio Siluk

**Affiliations:** a Federal University of Santa Maria, Innovation and competitiveness group, Production Engineering post-graduation program, Brazil; b University of Porto, Architecture and modes of inhabiting, Center for studies in Architecture and Urbanism, Portugal

**Keywords:** Smart city, Internet of things, Technological applications, Bibliometric analysis, Systematized literature review

## Abstract

The increasing urbanization in a poorly planned way accentuates the imbalance between the population's needs and the organized development in urban spaces. The present study is based on the development of a situational diagnosis in the scope of a smart city, for the contextualization of potential opportunities for actions and innovation strategies in urban spaces. This article presents a literature overview covering the innovative actions developed in the scope of smart cities in scientific publications. Furthermore, the scope of the study is identifying innovation initiatives in the performance of actions and solutions for urban spaces. A literature review was developed supported by mappings, couplings, and diagrams, through the use of *VOSViewer* and *SciMat* software, and 115 articles were selected and analyzed, considering the articles based on the criterion of the coefficient of the number of citations concerning the year of publication. In the literature overview developed, it was found that the research within the scope of smart cities has been deepened over the years, with the evolution of the number of words related to the theme in the period from 2014 to 2021, as the advance in the number of publications from 2018 is noticeable, which highlights the increase in popularity regarding the topic, as well as its current relevance. The study identified thematic axes with an emphasis on technology and innovation, environment, urbanism, energy, governance, mobility, and accessibility. The results contributed by assembling innovative smart city actions and practices in an interrelated way with technology, innovation, and market-oriented constructs aimed to reach urban demands, as well as the development of innovative solutions between public institutions and business organizations to integrate urban spaces.

## 1 Introduction

The urbanization process occurs unevenly over large territorial extensions around the world [1]. The increase in population density in urban areas, as well as the concentration of activities in cities resulting from urbanization, has created challenges for people's quality of life. The agglomeration of people in the urban environment triggers several problems related to transport, sanitation, energy, education, housing, and the generation of environmental impacts, among other obstacles to urban development.

In recent decades, the process of global urbanization has shown a constant evolution, with a substantial increase in the population in urban areas, which has been a constant concern, given the various impacts resulting from this growth. Based on United Nations records, in 1950, 30% of the world's population lived in urban areas, a proportion that evolved to 55% in 2018 [2].

Urbanization, which typically occurs in an unplanned way, generates an imbalance in the supply of the population's needs and results in disordered development in urban centers. A balance is reached when there is integration between the systems of assistance to the population, through actions that facilitate the exchange of information and the integration of data between urban mobility systems, public security, education, and health, and therefore the concept of smart cities emerges [3].

The use of technologies to improve urban management has grown over the years, with emphasis on the areas of energy efficiency, urban mobility, environmental management, and public safety. The term “smart city” was first brought to the scientific scenario by Ref. [4], highlighting the contribution of technological development to the creation of sustainable solutions, through the development of public and private sector initiatives focused on infrastructure for economic growth, market diversification, and global competitiveness.

Smart cities are innovative urban spaces characterized by the construction of infrastructure solutions that enable, for example, energy optimization, improvements in urban mobility, and minimization of waste generation to the environment. All these actions are based on the concentration of efforts in urban planning, based on the collaboration of people and organizations [5,6]. In this alignment, the theme has economic and social relevance, in line with [7], given the proposition of implementing smart initiatives, through innovation actions.

The proposition of using technologies and strategies with a focus on improving the quality of life and efficiency in resource management is aimed at the challenge of keeping up with the increase in urbanization and providing opportunities for the systemic and integrated management of human and material resources in urban spaces. Additionally, cities on several continents are seeking to develop sustainable actions, through technological tools and intelligent solutions aimed at optimizing urban living spaces, considering social, environmental, and economic aspects. The search for these actions, related to the improvement and greater efficiency in urban services aimed at incremental improvements and focusing on systemic and interactive spaces, promotes significant changes in living environments in cities [8].

Thus, smart cities, through technological solutions, involve, in essence, concern for the well-being of citizens, such as infrastructure for education and the search for knowledge, quality, and interactivity of urban services, mobility, and security [9]. Zanella et al. [10] add the application of the Internet of Things (IoT) paradigm to an urban context is of particular interest, since it responds to many demands for the use of Information and Communication Technologies (ICT) solutions in the management of public affairs, to contribute to the optimization of smart cities actions. In the field of technology, Low Power Wide Area (LPWA) networks envisage a new type of technology for communication in urban spaces, with the ability to offer affordable connectivity to low-power devices distributed over large geographical areas. In conjunction with the IoT, LPWA technologies complement or outperform conventional short-range cellular and wireless telephony technologies for various emerging applications within smart cities [11]. Ahvenniemi et al. [12] claim technologies applied in the context of urban spaces need to be smart, lean, integrated, economical, and resource-efficient, and must have an impact not only on environmental sustainability goals but also on social and economic sustainability.

The smart city is associated with the expectation of a better quality of life in urban environments through the relationship among local entities, companies, and citizens. Similarly, smart cities seek to develop the ability to interact with society and to provide citizens with intelligent services that help in their day-to-day life [10,13]. Therefore, the main objective is to present a literature overview of innovative actions developed within the scope of the smart city in scientific publications.

The main novelty is related to the research gap covered in this article. As the present study is a literature review article, the scope is to search relevant scientific publications, present contributions to the academic literature, contemplate technological solutions implemented to reach urban demands as well as the most relevant thematic axes in the context under study, the target audience involved in the development of smart cities initiatives, and strategic actions.

The present study is structured into five sections. Section 2 contemplates the review protocol constructed for the systematized literature review. Section 3 includes the descriptive analysis performed. Section 4 discusses perspectives of innovation and business, target audience, thematic axes, and technological applications. Section 5 contains the conclusion regarding the study developed.

## 2 Review protocol

The literature review was conducted through a research protocol conducted in three stages, based on the PRISMA 2020 statement [[14], [15], [16], [17]] and in the literature research developed in Refs. [[18], [19], [20], [21]]: (a) identification; (b) screening and included articles; and (c) descriptive analysis and perspectives. In the identification stage, the approach is defined through the design of the research protocol, study objectives, and review questions. The screening and included articles stage consist of deciding on the appropriate sources to select and extract information from the articles, through specific criteria and database search. Finally, the last stage includes the descriptive analysis, as well as perspectives and interpretations of the information found.

During the identification stage, the objective presented in Section 1 was developed, opening possibilities to elaborate four research questions: RQ1: How is state of the art for smart cities*?* RQ2: What are the main technological and innovation actions developed which are applicable in the urban context? (Influencer aspects), RQ3: What thematic axes are relevant in the context of smart cities? (Scope delineation), and RQ4: Who is the focal target audience of the actions developed in smart cities? (Typology). These questions guided to the string ("smart cit*") AND ("technol*" OR "innov*") AND ("businn*" OR "perform").

In the screening and included articles stage, initially, the research sources were selected with the use of two databases: *Scopus* and *Web of Science*. The review was developed in October and November 2021 including only articles published in scientific journals (not conference articles or technical papers, for example) in the English version from 2014 to 2021, search by “Title, abstract or keywords” (*Scopus*) and “topic” (*Web of Science*). The literature review was not conducted for the period before 2014 to restrict the search size, given the need to assist the target audience involved in the development of smart city initiatives by showing recent technologies, innovation actions, and thematic axes for the development of the solutions in the smart city scope.

With 2516 articles (1263 from *Web of Science* and 1253 from *Scopus*) being located in the macro sphere of the research. After excluding duplicated articles and eliminating articles with titles and abstracts not related to the theme, the number of articles was reduced to 1559 articles. Next, the selection criterion was developed based on the quotient between the number of citations and the time of publication of each article. For example, if an article was published in 2014 and until 2021 has 100 citations, then the quotient is equal to 100 divided by 7, which is equal to 14.28. With the quotient selection criterion defined, a total of 115 articles with the highest reference index were selected, representing approximately 7% most represent articles from the list of 1559 articles previously reduced. Considering the interest in conducting a literature review to reach urban demands as well as the most relevant thematic axes in the context under study, the target audience involved in the development of smart cities initiatives, and strategic actions, literature review articles were considered for the quotient selection.

Finally, the descriptive analysis and perspectives stage was divided into two parts. The descriptive analysis in Section 3 was developed considering all 1559 articles, while the perspectives were discussed and interpreted in Section 4 using only the 115 articles filtered with the quotient selection criteria.

## 3 Descriptive analysis

The bibliometric analysis referring to the 1559 selected articles was developed to determine the quantitative and qualitative contributions to the scientific field addressed. Three main methods were explored: citation analysis by articles, bibliographic coupling, and citation analysis by authors. Then, an analysis was developed regarding the main words of the theme, as well as their evolution over the years. The groupings formed for each method are unrelated to the groupings for the other methods. The VOSViewer 1.6.17 software [22], and the SciMat software [23] were used to construct and visualize the mappings and also as a tool for text exploration, with the articles arranged in the *Scopus* and *Web of Science* databases as a scientific reference.

**Fig. 1:**
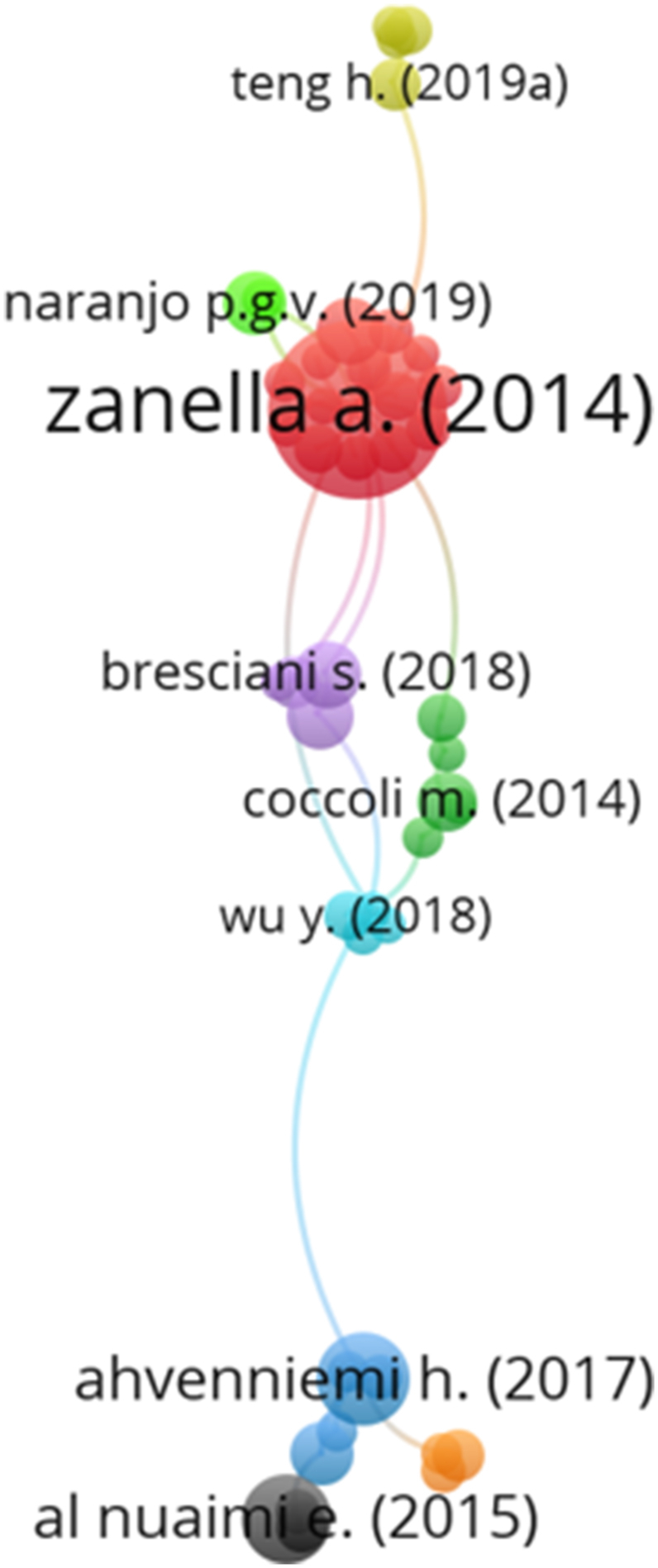
Citation analysis map by article.

Initially, citation analysis by articles was performed, which measures the connection between articles based on the number of times that an article was cited. Only articles with at least 25 citations in the *Scopus* and *Web of Science* databases were considered for the study, thus resulting in 221 articles analyzed. The minimum number of articles needed to form a grouping was defined as two. Only 45 articles presented some degree of connection, which can be seen in the analysis map (Fig. 1). Nine groupings of connections among the articles were identified. Grouping 1 (red) presented behavior involving different applications of the IoT in smart cities, centered around [10]. The grouping presents articles related to information and communication technology [24]; industry 4.0 [25]; waste management [26]; vehicular communication [27]; use of drones and internet for public safety [28]; intelligent and connected transportation systems (ICTS) [29]; energy efficiency [30]; IoT platforms [31]; crowd dynamics management [32]; intelligent environments [33]; indoor location [34]; electrified transport network [35]; and financial investment in information technologies and smart city systems [36].

Grouping 2 (green) is related to the use of technology in smart cities, addressing the adoption of services involving information technology in India's smart cities [37]; big data, and cognitive computing in the learning process [38]; virtual reality [39,40]; and a model related to the adoption of a variety of smart solutions in university environments to improve the quality of life and performance of both teachers and students [41]. Grouping 3 (blue) involves articles focusing on the sustainability of smart cities [12,42,43]; the acceptance of smart homes by students with a high educational level [44]; and the smart city attractiveness for tourists and residents [45]. The articles in grouping 4 (yellow) show the communication and spread of the IoT in smart cities by mobile vehicles [46], vehicles with sensor devices [47], using vehicular networks [48], and presenting a low latency communication scheme for wireless mobile sensor control systems [49].

In grouping 5 (purple) practical applications related to the development of smart cities involving the IoT [50,51]; barriers encountered in the development of smart cities in the context of India [52]; and an investigation into citizen participation in the smart city project [53] are presented. Grouping 6 articles (light blue) are related to data generation involving smart cities, highlighting the importance of digital data for the smart city policy [54]; presenting smart cities in the context of big data [55]; and developing data for smart cities based on the case of Catania [56]. The grouping also presents a literature review identifying the most and less popular themes involving smart cities [57]. In grouping 7 (orange), the articles address the transition from normal cities to smart cities, presenting a transformation framework [58], comparing the development patterns used in China [59], and obtaining quality data [60]. Grouping 8 (black) is related to the urban development of smart cities, using big data applications [61], promoting a vision for future smart sustainable cities [62], and implementing smart urban metabolism [63]. Finally, the articles in grouping 9 (light green) are related to fog-based architectures for IoT environments in smart cities [64,65].

**Fig. 2:**
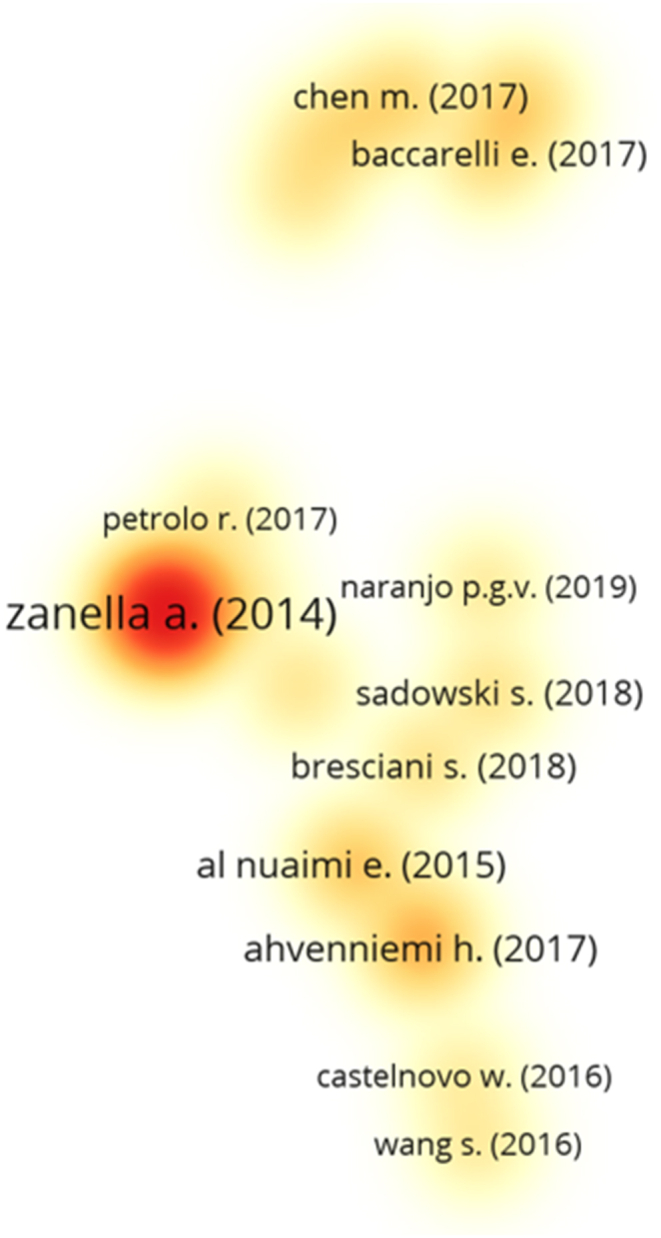
Bibliographic coupling map.

The similarity analysis of the articles was performed based on the number of shared references to visualize their degree of connection and the bibliographic coupling map. The size of the circles represents the total connecting force for each article and the intensity of the colors represents the total connecting force of the formed groupings. Darker color intensity represents similarity between articles of the same grouping. The groupings formed in the bibliographic coupling map are based on the number of references shared by articles of the same grouping. Altogether, 35 articles with at least 100 citations were considered for the study, and 20 of them presented some degree of connection, as shown in Fig. 2. Five groupings were identified. Grouping 1 (bottom position) consists of articles related to smart cities’ sustainability [12,43]; governance and policy of smart cities [66]; energy harvesting from urban environments [67]; and big data implementations in smart cities [61,68].

Articles in grouping 2 (left center position) address practical applications of smart cities [10,51,69]; research on the vision and paradigms of smart cities [70]; and a framework related to the analysis of large-scale data related to smart cities [71]. Grouping 3 (top left) presents a review of IoT applications [72]; a deep cognitive perspective [73]; an integrated framework that enables dynamic orchestration of network, cache, and computer resources [74]; and a review of the *narrow-band* IoT [75]. Grouping 4 (right center position) refers to IoT environments [64]; a comparison between four wireless technologies for an indoor location using the IoT [34]; and research addressing multinational corporations (MNCs) related to the development of knowledge management capabilities and information and communication technologies [50]. Finally, grouping 5 (top right position) is related to the smart vehicles present in smart cities [76]; intelligent electric vehicle charging [77]; and *fog-based* architectures [78].

**Fig. 3:**
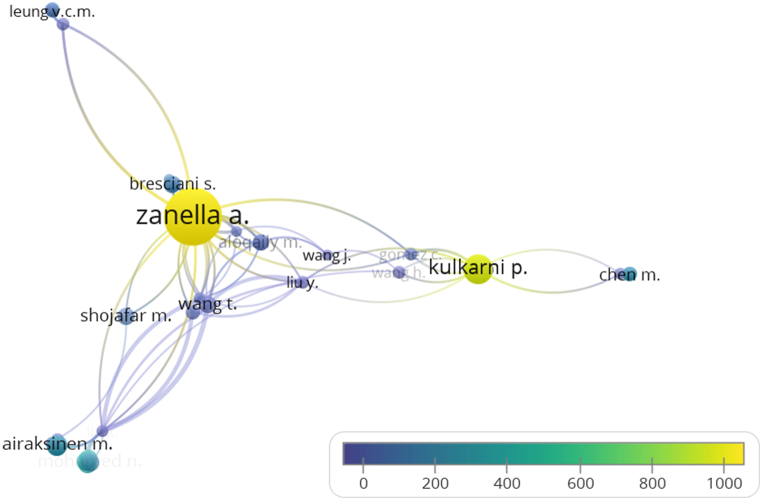
Citation analysis map by authors.

The verification of the researchers' network was developed to expand the citation analysis about smart cities. A total of 52 studies were selected considering researchers with at least 150 citations. The colors vary according to the average number of citations per year, with the lighter colors being related to authors with the highest average number of citations per year and the darker colors being related to authors with the lowest average number of citations per year. Also, the size of the circles varies according to the total number of citations. The map shown in Fig. 3 shows the connections among studies based on the number of times the articles were cited. The representativeness of Zanella's study stands out, presenting a high number of citations in the study's clipping area, through the relationship between smart cities and IoT, occupying a highlighted position by the identified interrelationships of related citations. In the second position, in terms of intensity, is the author Kulkarni, with an approach more focused on communication networks. Furthermore, other researchers in the area, which include in their research thematic alignments, were found to have intermediate amounts of citations, as identified in the established network.

**Fig. 4:**
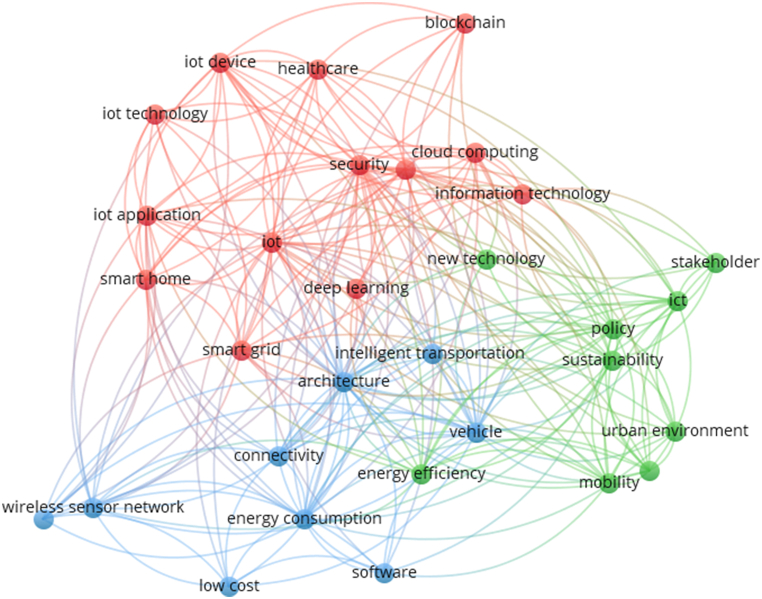
Co-occurrence map by title and abstract.

To verify the co-occurrence of words in the title and abstract of the selected articles, a co-occurrence mapping was created, in which the most used keywords in the title and abstract of the selected articles were identified. Each grouping is formed by the relationship between the words with the highest frequency of use, and the words with at least 30 (thirty) occurrences being selected. In addition, general words such as “idea”, “image”, “end”, “process”, “software”, and “web” were removed. Fig. 4 shows the co-occurrence map with the identification of the most used words. There are three clusters of keywords in the set of selected articles. In cluster 1 (red) the words are connected, having the IoT as the central axis. Cluster 2 (green) connects words, in which it is found that sustainability and policy (public management) stand out for the interrelationships established in the scope of scientific publications. In cluster 3 (blue), the words energy consumption and architecture had a higher frequency of use, which demonstrates the relevance of applications aimed at structural adaptations and energy efficiency in the scope of the reference articles for the study.

Following, the *SciMAT* software was used to analyze the trends of use of the words in the period under study, having as reference a minimum number of occurrences for each period, based on the respective number of documents found. For the period from 2014 to 2015, the minimum number of two occurrences was considered; for the period from 2016 to 2017, three occurrences; for the period from 2018 to 2019; four occurrences; and for the period from 2020 to 2021, five occurrences. The data analysis followed the following parameters: (i) unit of analysis: words; (ii) matrix type: co-occurrence; (iii) normalization measure: equivalence index; (iv) grouping algorithm: simple centers algorithm; (v) document mappers: core mapper and secondary mapper; (vi) quality measures: h index, average citations, and the sum of citations; (vii) measures for the longitudinal map: equivalence index.

**Fig. 5:**
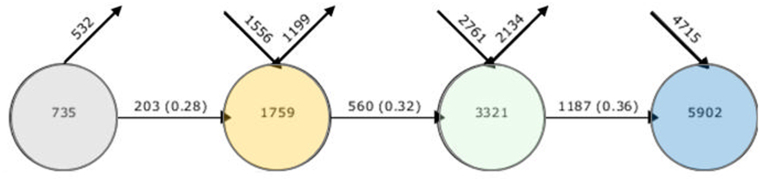
Movement of the main words by period.

To show the behavior of the words related to the research topic in each of the determined intervals: 2014–2015, 2016–2017, 2018–2019, and 2020–2021, the software *SciMAT* was used. The periods were divided in this order due to the research selection criteria, which consider the articles in the thematic area from 2014. Also, the last period involves articles from 2020 to 2021. Fig. 5 shows the movement of the main words in the period from 2014 to 2021. To obtain the analysis of words by period, the articles’ data were exported from the *Scopus* and *Web of Science* databases in the “*.ris*” format and subsequently imported into the SciMAT software. Articles related to the topic “smart city” presented 735 main words in the first period, reaching up to 5902 main words in the last period, which indicates an increase in popularity over the years and highlights its current relevance.

**Fig. 6:**
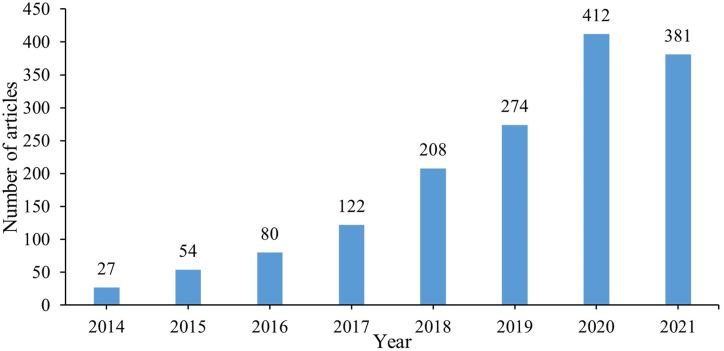
Number of articles by year.

The percentage of words that remain in use between periods, represented by the horizontal arrow, increases progressively from 28% between the first and second periods to 32% between the second and third periods, and 36% between the third and last periods. This fact shows that part of the research related to the theme is being deepened every year. Furthermore, the number of words discarded at each period, represented by the right diagonal arrow, is lower than the number of new words inserted into the theme, represented by the left diagonal arrow, identifying an increase in the scope of the theme. Fig. 6 shows the number of articles in each period analyzed. The number of articles shows the evolution of the theme in recent years, which justifies the period selected to be studied. There is a significant growth of publications in the thematic area from 2018 and, after 2020, an expansion with a high amplitude of scientific productions.

**Fig. 7:**
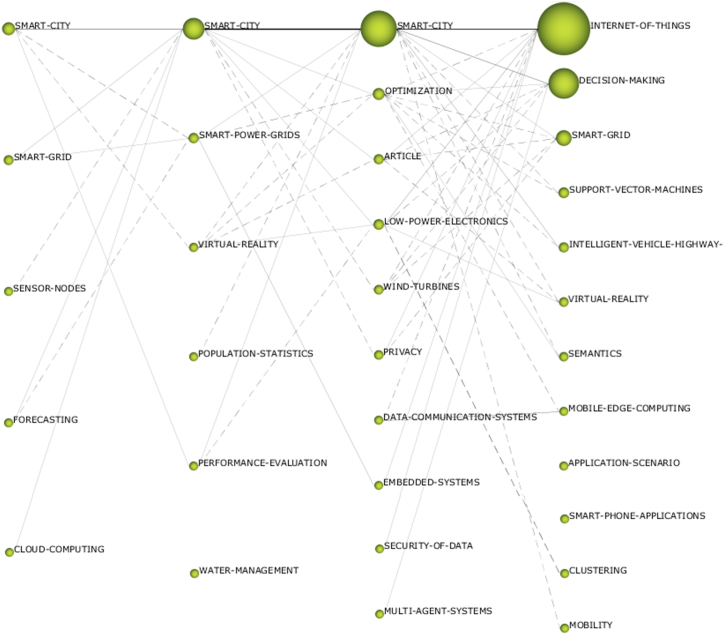
Relationship among the main words.

The main words used in the period are also relevant for checking trends and connections with each other, identifying categories of words, and their interrelationships. Fig. 7 shows the relationship among the main words used in the reference articles by period. Two categories of lines connect the circles of the different periods: continuous lines and dashed lines. Solid lines indicate a strong relationship between words, and dashed lines indicate weaker relationship trends. Between the periods analyzed, “smart cities” and “internet of things” were the words with the greatest connection, having a strong connection with each other. These words also have a continuous connection with other words or terms, with emphasis on “decision-making”, “optimization”, and “smart-grid”*,* which shows the strong relationship with the need for decision-making focused on optimization, through action in smart grids.

**Fig. 8:**
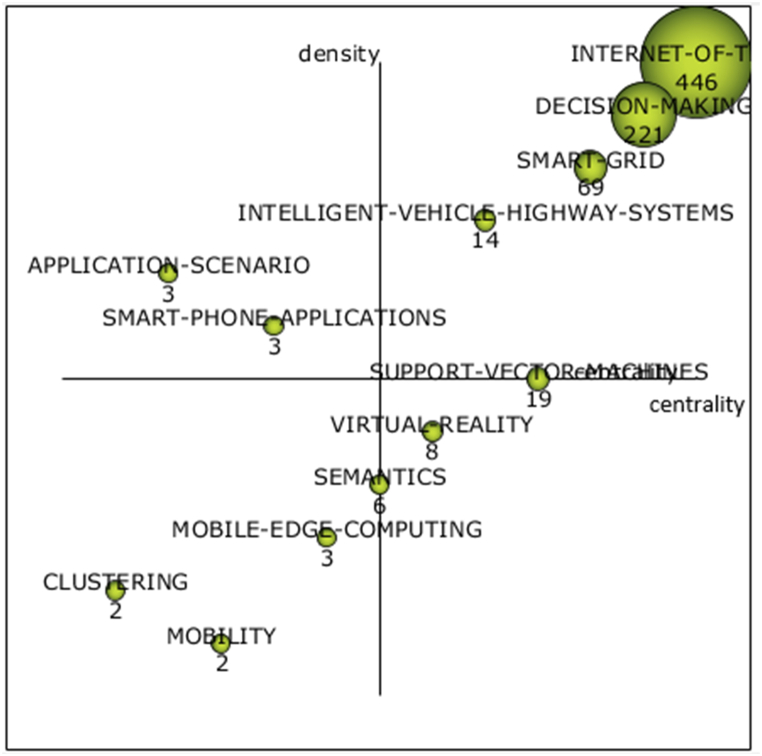
Strategic diagram.

In the search to identify the relationships between the main and secondary terms in the studies, a strategic diagram was developed, which demonstrates the centrality and density of the words in the articles analyzed in the period. Further to the right of the diagram, the more central the term is considered, and the higher it is, the denser. The grouping network (right) presents which words are linked to the main term and shows how strong the relationships between the secondary terms and the main term are in the most current period (2020 and 2021). Therefore, the terms represented in the first quadrant of the diagram are considered the most relevant for the research area. Fig. 8 shows the strategic diagram with the identification of the centrality and density of the words that integrate the articles. In the period from 2020 to 2021, the centralizing term “internet of things” was mentioned 446 times. Words like “decision-making”, “smart-grid”, and “intelligent vehicle highway systems” are densely researched. The words and terms that have the most relevance to the research topic are “smart city”, “energy efficiency”, “deep-learning”, and “block-chain”. Among words and terms with little connection with the term “internet-of-things”, there are “performance evaluation”, “cost reduction”, and “privacy and security”, which may represent possibilities for new research to be conducted in the future.

## 4 Perspectives

With the 115 articles selected, the theme underwent new conceptions and is strongly associated with cities that apply the use of information technology for sustainable actions that promote an improvement in people's quality of life. Of the articles that integrate the scope of the study, most were published between 2018 and 2019, being 52 articles, 45.22% of the publications, and when considering together with those published between 2020 and 2021, the total reaches 82 articles, which represents 71.31% of the studies in the period. Most of the publications in the study period were from 2018, in alignment with the proportion of the general production in the thematic, as represented in Fig. 6.

In the course of the descriptive analysis of the publications of the 115 selected articles, perspectives were delimited for the detailing of the findings by bibliographic coupling, aiming to contemplate the technological applications, the thematic axes, the target audience of the studies, and innovation and business, to seek a better understanding in the context of the research field. Technological applications are present in many actions and strategies of smart cities. This interrelation is highlighted within the scope of the area under study.

### 4.1 Smart cities and technological applications

In the field of study, the interrelationship between smart cities and technology, through the development of various intelligent actions and strategies with technological applications, is incisive. According to Al Nuaimi et al. [61], smart cities use multiple technologies to develop actions to improve health, transport, energy, education, and services, aiming to provide greater comfort to citizens. Thus, technological applications through the IoT, communication networks, computing, and big data are highlighted from the selected articles the frequency of applications identified, with representation above 10.00% in the selected articles, individually or simultaneously, as shown in Table 1. Regarding technological application, applications in the context of the IoT stand out, covering 71 articles, which represents 61.74% of the material under study. In second place are applications in the area of communication networks, which appear in 51 articles, representing 44.35% of the analyzed productions. Thereafter, there are applications in the area of computing and big data, in 22 and 15 articles, respectively, which represent 19.13% and 13.04% of the scope of the articles.

**Table 1:** Technological applications.

Application	Quantity	Frequency
Internet of things	71	61.74%
Communication networks	51	44.35%
Computing	22	19.13%
Big data	15	13.04%

The articles present a centrality between smart city and IoT, given the development of applications and implementation of IoT solutions in 71 of the 115 selected articles, which represents 61.74% of the articles analyzed. According to Asghari et al. [72], the IoT elevates the benefits to human life through the environment in which intelligent services are provided, mainly for the development of activities in any place and time. The facilities and services are conveyed through applications conducted in environments mediated by the IoT. The most cited study in the field of smart cities*,* published in Ref. [10], discusses the relationship between smart cities and the IoT as a paradigm of recent communication that, when applied in urban environments, is capable of incorporating a large number of different and heterogeneous systems for the development of services and communication and information technology solutions in management and urban planning. Bresciani et al. [50] address the application of the IoT related to innovation in business environments. In smart cities*,* the development of ICT to support intelligent services to citizens is prioritized. The IoT, on the other hand, is aimed at the implementation of technological tools, generating opportunities for companies to innovate in the development of applied solutions. In this context, companies seek to develop initiatives and projects, aiming at the qualification of services and the implementation of new IoT-based devices to meet the demands of citizens through projects, actions, and businesses.

Among the IoT applications in smart cities, the growing development of online applications with computing, communication, and intelligence resources is relevant. Aazam et al. [79] highlight the applications aimed at assisted living environments (ALE), virtual reality, augmented reality, and intelligent vehicular communication, highlighting the interaction between the ones involved to achieve the ultimate goal of applications in the context of smart cities. Regarding the analyzed articles, 51 (44.35%), address the applications of communication and information networks. According to He et al. [74], in the smart cities ecosystem, large amounts of data and information will be replaced by devices and networks, with emphasis on functionality through software and virtualization of network functions, which allows the abstraction of physical resources in networks and flexible sharing of resources by several users.

Applications in the area of computing are a theme found in 22 articles, that is, 19.13% of publications. According to Zhou et al. [80], computing provides opportunities for applications in various types of technologies and scenarios, given that, with rapid technological development, a variety of new smart devices have emerged and were widely applied in the context of smart cities, awakening the exploration of new paradigms in the academic community and the business environment. Big data applications were identified in 15 articles. According to Al Nuaimi et al. [61], the available data are generated from various sources, resulting in the formation of what is currently known as big data. Data sources are all around us in many places: smartphones, computers, environmental sensors, cameras, geographic positioning systems (GPS), and even people. There is a variety in the potential use of big data as a strategic analytics engine for deeper insights through intelligence and data mining in urban environments. For Ahvenniemi et al. [12], the development of the smart city concept, in the search for environmental sustainability, runs through the implementation of applied technologies. The growing interest in the concept of a smart city and the need to solve the challenges related to urbanization lead to diverse private and public investments in technology applications and the deployment of big data systems. For Al Nuaimi et al. [61], big data systems make it possible to store, process, and exploit information from smart city applications to produce information and improve different services, with emphasis on applications in health, energy, transport, environment, security, education, and smart governance.

In the articles analyzed, the big data system applications are diversified, with emphasis on applications for data sharing, application development, exploitation of energy services, urban infrastructure monitoring, and health services monitoring. In addition, they address structured applications for the development of sustainable city models, security strategies, and applications in artificial intelligence, in the search for convergence of integrated data, through electronic devices and technological applications.

### 4.2 Thematic axes

In the development of the study, 12 categories of thematic axes were identified, which appear individually or integrated. In Table 2, the categories that were most frequently addressed are listed, with a minimum percentage of 10% in the scope of the study. Based on the categorization, the 115 selected articles, address, directly or indirectly, the thematic axis of technology and innovation. Among the other categories, the applications that have the themes of environment, urbanism, energy, governance, mobility, and accessibility as a guiding axis stand out. Zanella et al. [10] highlight the development of smart cities designed and supported by urban IoTs, based on the exploration of technology and innovation, in the planning of value-added services, through technical solutions and best practice guidelines for citizens, companies, and management of urban spaces. In this same alignment, according to Qureshi et al. [81], new communication technologies have played a vital role in transforming traditional urbanization into intelligent and comfortable environments for citizens. With the integration of new standards and systems, smart cities faced several challenges related to technologies, systems management, and scalability in proposing appropriate actions for their living spaces.

**Table 2:** Thematic axes.

Description	Quantity	Frequency
Technology and innovation	115	100.00%
Environment	17	14.78%
Urbanism	15	13.04%
Energy	13	11.30%
Governance	13	11.30%
Mobility and accessibility	12	10.43%

The scientific productions present an integrated characteristic, 14.78% of the articles contemplate applications in the area of the environment, with emphasis on sustainability and environmental management. As for the environment, cities play a fundamental role in preservation actions, whose implementation of new smart technologies is seen as a fundamental factor to reduce greenhouse gas emissions. These technologies need to be integrated, cost-effective, and resource-efficient aimed not only at environmental sustainability but social and economic sustainability [12]. The thematic axis urbanism appears in 13.04% of the studies, focusing on current urban trends and the improvement in people's quality of life, as well as the use of technologies to give more practicality to the urban environments' routines. According to Ref. [58], smart cities, through the incorporation of technologies and the IoT, promote urban development and improvements in city operations and routines, as well as intelligent analysis to optimize services, production, and usability in urban environments.

In 11.30% of the articles, applications focused on the energy theme were found, with an emphasis on alternative forms of energy and on energy optimization and efficiency. Built environments are responsible for a significant volume of energy consumption. For Jain et al. [82], in the United States, 40% of energy consumption occurs in built environments, a proportion that is also found in several countries. In dense urban areas, such as around New York City, urban buildings account for more than 90% of electricity use and 75% of greenhouse gas emissions. Therefore, characterizing, modeling, and predicting energy consumption is crucial for urban areas to reduce their energy consumption. Modeling and forecasting energy demand provides opportunities for numerous energy management and efficiency applications, through urban energy infrastructure planning.

The governance category was also addressed in 11.30% of the publications, with emphasis on the development of collaborative governance, urban policies, and digital participation applications. Al Nuaimi et al. [61] address the concept of a smart city associated with the city that invests in improved governance of technologies and participatory processes to define appropriate investments in public services, ensuring sustainable socioeconomic development, a better quality of life, and intelligent management of natural resources. According to Bibri and Krogstie [62], governments of technologically advanced nations face significant challenges due to issues engendered by urban growth. Problems include increased energy consumption, pollution, waste disposal, inefficient management of urban infrastructure, ineffective planning processes and decision-making systems, social inequality, and socio-economic disparity. These challenges reinforce the need for smart city actions and strategies in the transformation of different areas of human life, regarding the incorporation of sustainable development goals in their strategies.

The mobility and accessibility category was found in 10.43% of the studies, contemplating the optimization of locomotion, intelligent mobility, and adaptations in urban environments. According to Qureshi et al. [81], smart mobility encompasses all types of transport within cities. The transport system is considered one of the main pillars of mobility in urban spaces. In cities, traffic congestion is a significant problem, as are complex road structures and poor traffic control systems. In smart cities, applications focus on traffic management and control systems, such as security and infotainment applications for the coexistence of drivers and passengers, and ad hoc vehicle networks, which provide the opportunity to exchange information about traffic and urban road conditions in real time. The tracking and location of vehicles is also a domain of intelligent mobility, with the use of communication and navigation systems, which provides traffic monitoring and management of the transport system.

### 4.3 Target audience

The productions in the thematic area present a broad and diverse target audience, with studies developed under the academic focus, as well as studies that contemplate integrated applications in the scope of companies. An interrelation between the areas of action stratified in the two categories of the target audience was identified for the alignment between the conceptual part of the theme and the applied development of technologies that meet the demands in the study area. According to Al Nuaimi et al. [61], as studies advance and more research and development efforts are applied to the design of smart cities, the problems and challenges of urban spaces are addressed with greater amplitude in the search for solutions. The expected result is that more cities will develop smart applications, focusing on residents’ quality of life. Such applications should consider the diverse requirements of the smart city (physical, social, and technological) in an integrated and transversal way, through a holistic approach that will help identify the demands and appropriate solutions for smart cities.

Bibri and Krogstie [62] highlight the importance of research in the context of smart cities, given perspectives that cover smart actions and practices, as well as advances in sustainability analyses. The study reinforces that sustainable smart urbanism involves the development of urban intelligence functions as an advanced form of decision support, which represent new conceptions of how smart cities act and use science in creating new forms of urban simulation and optimization and forecasting methods. Ahvenniemi et al. [12] mention that the growing interest in the theme and the need to solve the challenges related to urbanization provides opportunities for private and public investments in the development and implementation of technology, which requires the effective participation of researchers and companies, in an integrated and associated way, in the search for technological implementations.

### 4.4 Innovation and business

Within the framework of smart cities, technological innovation is essential for the development of applications and solutions aimed at the main challenges of urbanization, aiming to improve city management and social coexistence. In the view of Al Nuaimi et al. [61], the use of technological resources and the development of applied tools encourages collaboration and communication between the different entities of a smart city, promoting innovation and the construction of collaborative and creative application solutions for areas such as education, health, energy, industry, environment, and security. Zanella et al. [10] highlight the use of communication technology solutions integrated with the IoT in the urban context, in the search for the best quality of services provided to citizens, collaborating with the development of actions for the active participation of people. These actions also contribute to stimulating the creation of new services in various areas, such as transportation and parking, surveillance and maintenance of public areas, preservation of cultural heritage, garbage collection, healthcare, and educational institutions.

For Bibri and Krogstie [62], smart cities need to develop and implement innovative solutions and sophisticated approaches supported by cutting-edge technologies and innovative scientific knowledge, aimed at improving the sustainability, efficiency, resilience, equity, and living conditions of citizens. According to Kumar et al. [83], current urbanization requires strategies and planning for the modernization of urban life, through advanced ICT solutions. These actions promote the development of business activities focused on digital technology infrastructure for smart cities, a demand that is directed towards the production and availability of precise technology, which also contributes to companies acting more competitively. In Manchester, cutting-edge technologies have been developed for business, based on the IoT, innovation laboratory, and to facilitate access to open data to design new services. In Seoul, virtual shopping malls have been set up at bus stops so citizens can order online while waiting for the bus. Similarly, the smart city of Birmingham has developed i-tech hubs to develop e-businesses and innovation.

According to Qureshi et al. [81], smart living is a broad area where various services are integrated to improve the quality of life, contemplating safety, health, education, tourism, smart buildings, and disaster management. They highlight a service developed within the scope of public safety, with a system designed with interactive voice response, where people report and receive feedback on any concern related to safety. Another aspect of intelligent life is health care, where real-time care systems have been designed for the elderly, athletes, and other patients to monitor heart rate, brain functions, physical movement, and other parameters relevant to health. Medical centers are connected with wearable devices to serve patients in their homes and hospitals. They also address smart actions in the support ecosystem for tourism services, which provide service support to visitors, promote local businesses, and play a significant role in the economic growth of cities.

Given the scenario under study, for the management of urban spaces, it is necessary to adapt processes and implement planning strategies, in an integrated way with technological advances, and with perspectives of actions in a network of educational institutions, researchers, companies, and public managers.

### 4.5 Future perspectives

The use of urban spaces contemplates a set of actions regarding its planning and optimization. Technological solutions are necessary for cities to innovate in the organization of their urban spaces, such as the integration of public transport networks, traffic light control, automated traffic signaling, traffic plan for urban areas, geospatial monitoring system, and parking lots with occupancy sensors. Additionally, there are service-user integration actions, with the use of software and applications that make it possible to carry out sustainable urban planning actions, with the effective participation of users. The IoT is intensely present in many aspects of human life, such as cities, homes, educational institutions, business organizations, agricultural environments, hospitals, and health centers. Numerous features such as producing/consuming data and online services improve daily life and activities around the world through the context of IoT. As the desires of users grow, innovative applications are being provided to monitor, manage, and automate human activities [72].

Within the scope of smart cities and the development of these actions and technological solutions, an integrated system of cooperation is necessary, through the interaction between government, society, companies, and educational institutions, in the search for the implementation of intelligent actions and strategies in urban spaces. In this sense, it is evident that, for the development of new perspectives in the field of smart cities, it is necessary for the involvement of educational institutions, acting in technical qualification and academic training, as well as a partnership with business organizations for technological applications aimed at proposing products and services in the search for an innovative environment. Thus, the business environment is highlighted as a driver of actions aimed at implementing an innovation ecosystem and proposing applications of intelligent solutions that meet local demands with added value to smart cities, including operational efficiency, provision of services to innovation, and market strategies.

In proposing actions, through business solutions that are aligned with the needs of citizens and the particularities of the implementation region, the following questions are presented.

- How to develop smart solutions, within the business scope, that meet the demands of society's interest and contribute with appropriate technological solutions for smart cities?
- What indicators are appropriate to assess the technological criteria applied to the innovative context of a smart city?
- In what way can collaboration occur in the search for innovative integrated solutions and intelligent actions in urban spaces?
- How to design the thematic axes in the context of smart cities, within the scope of innovation management, from the perspective of ideation and applications aligned with market demands?

Given the perspectives in the thematic area, there are limitations, mainly in terms of obtaining data and indicators for the development of applied and qualified studies within the scope of the municipalities. The implementation of actions for urban planning requires an integrated set of public and private data, which are strategic for decision-making. For the performance of companies, the availability of private data is essential for the proposition and implementation of actions in the area of smart cities. Obtaining data usually present costs, which are sometimes made possible through partnerships and agreements, which are necessary to provide opportunities for actions aimed at the challenges of urbanization.

## 5 Conclusion

- The main objective of the present study is to present a literature overview on innovative actions developed within the scope of the smart city in scientific publications, demonstrating the context of the theme in the period 2014 to 2021;
- A total of 2516 articles were identified in the *Scopus* and *Web of Science* databases, with 115 articles selected with the highest quotient between the number of citations and the time of publication;
- The literature overview showed the thematic relevance in the current technological and innovative scenario of urban spaces;
- Zanella et al. [10] is the article with the highest number of citations by the time of publication, highlighting the relevance of the smart city concept to public management, which can act as catalysts for smart actions to improve the cities environment;
- Population growth and urbanization have intensified the development of actions aimed at reconciling the citizen's lifestyle with the environment, economic issues, and governance actions;
- The use of communication technologies has a vital role in transforming traditional urbanization into smarter and more comfortable spaces for the citizen, with challenges related to how to develop scalable and secure data management systems;
- Regarding technologies, the internet of things and communication networks can be the basis for the development of unified information and communication technology platforms on an urban scale, unlocking the potential of a smart city. Also, with computing and big data technologies, retaining structured data from different sources is possible, allowing the possibility of validating the data necessary for decision-making and services planning;
- The thematic axis of technology and innovation is contemplated, directly or indirectly, in all investigated articles. Applications focused on the thematic axis environment, urbanism, energy, and governance are also highlighted;
- The planning of innovation actions to seek the implementation of scalable technologies for smart cities require the involvement of different actors in an organized and organic way. The target audience for research on smart cities can be classified into two categories, researchers and companies, with the identification of an interrelation necessary for the development of smart cities technologies to reach the market demands and citizens' needs;
- In this way, this research contributed by assembling innovative smart city actions and practices in an interrelated way with technology, innovation, and market-oriented constructs aimed to reach urban demands.

## Data availability statement

The authors confirm that the no extra supporting data are available for this article.

## CRediT authorship contribution statement

**Elizeu Jacques:** Conceptualization, Data curation, Formal analysis, Investigation, Methodology, Writing – original draft. **Alvaro Neuenfeldt Júnior:** Funding acquisition, Investigation, Supervision, Writing – review & editing. **Sabine De Paris:** Methodology, Validation, Visualization, Writing – review & editing. **Matheus Francescatto:** Formal analysis, Methodology, Writing – review & editing. **Julio Siluk:** Conceptualization, Project administration, Validation.

## Declaration of competing interest

The authors declare the following financial interests/personal relationships which may be considered as potential competing interests:Alvaro NEUENFELDT JÚNIOR reports financial support was provided by 10.13039/501100003593National Council for Scientific and Technological Development. Elizeu JACQUES reports financial support was provided by Coordination of Higher Education Personnel Improvement. Matheus FRANCESCATTO reports financial support was provided by Coordination of Higher Education Personnel Improvement. If there are other authors, they declare that they have no known competing financial interests or personal relationships that could have appeared to influence the work reported in this paper.
